# Impact of bicuspid aortic valve orifice orientation on simple ascending aorta replacement

**DOI:** 10.1016/j.xjse.2025.100058

**Published:** 2025-05-29

**Authors:** Francesco Giosuè Irace, Ilaria Chirichilli, Andrea Salica, Raffaele Scaffa, Ruggero de Paulis

**Affiliations:** a Department of Cardiac Surgery and Heart Transplantation, San Camillo Forlanini Hospital, Rome, Italy; b Department of Cardiac Surgery, European Hospital, Rome, Italy; c Cardiac Surgery, UniCamillus International University of Health Sciences, Rome, Italy

**Figure:**
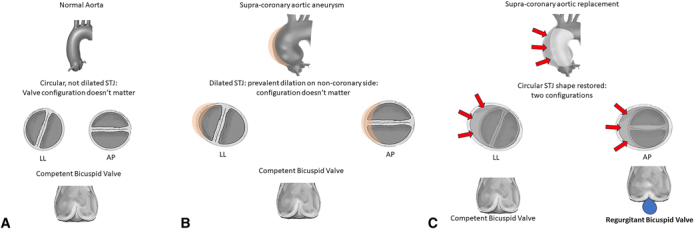
BAV orientation relative to the STJ. Normal (A), aneurysmatic (B), and after aorta replacement (C).

**Central Message**

Ascending aorta replacement in BAVs may interfere with aortic root geometry, depending on the orientation of the valve orifice. Leaflet prolapse may be induced and cause valve regurgitation.

In supracoronary aorta replacement, the sinotubular junction (STJ) divides the normal-size aortic root from a dilated ascending aorta. If the STJ is not involved in the dilatation of the aorta and maintains a normal diameter, the aortic valve is usually competent, and the ascending aortic replacement has no effect on the valve function regardless of whether it is a tricuspid valve or a bicuspid aortic valve (BAV). Considering a patient with a normally functioning valve, the progressive STJ dilatation will have different effects on a tricuspid valve or BAV. In the presence of a tricuspid valve, the STJ dilatation will progressively pull on the 3 commissures generating a tethering effect on the leaflet free margins, which in turn will progressively open a central orifice on the valve with consequent central regurgitation. Restoration of normal STJ diameter by replacing the ascending aorta will restore valve competence, provided that the proper geometric relationships among the 3 commissures are correctly maintained.

In the presence of a BAV, the effect on the valve is highly dependent on the commissural orientation (CO) and its rotational position with respect to the heart. Indeed, slight rotation of the aortic root could alter the orientation of the commissures toward the major diameter of the aneurysm.^1^ When the ascending aorta is dilated, the STJ is usually involved along the great curve of the aorta toward the noncoronary sinus, resulting in a slightly ellipsoid shape.

This ellipse is more pronounced when the CO is perpendicular (or nearly perpendicular) to the axis of dilatation (Figure 1, *A*). In this case, reducing the aortic diameter and restoring a circular shape by implanting a tubular Dacron graft do not significantly affect valve function, because the commissural “pillars” hold de facto still in the same position.

**Figure 1:**
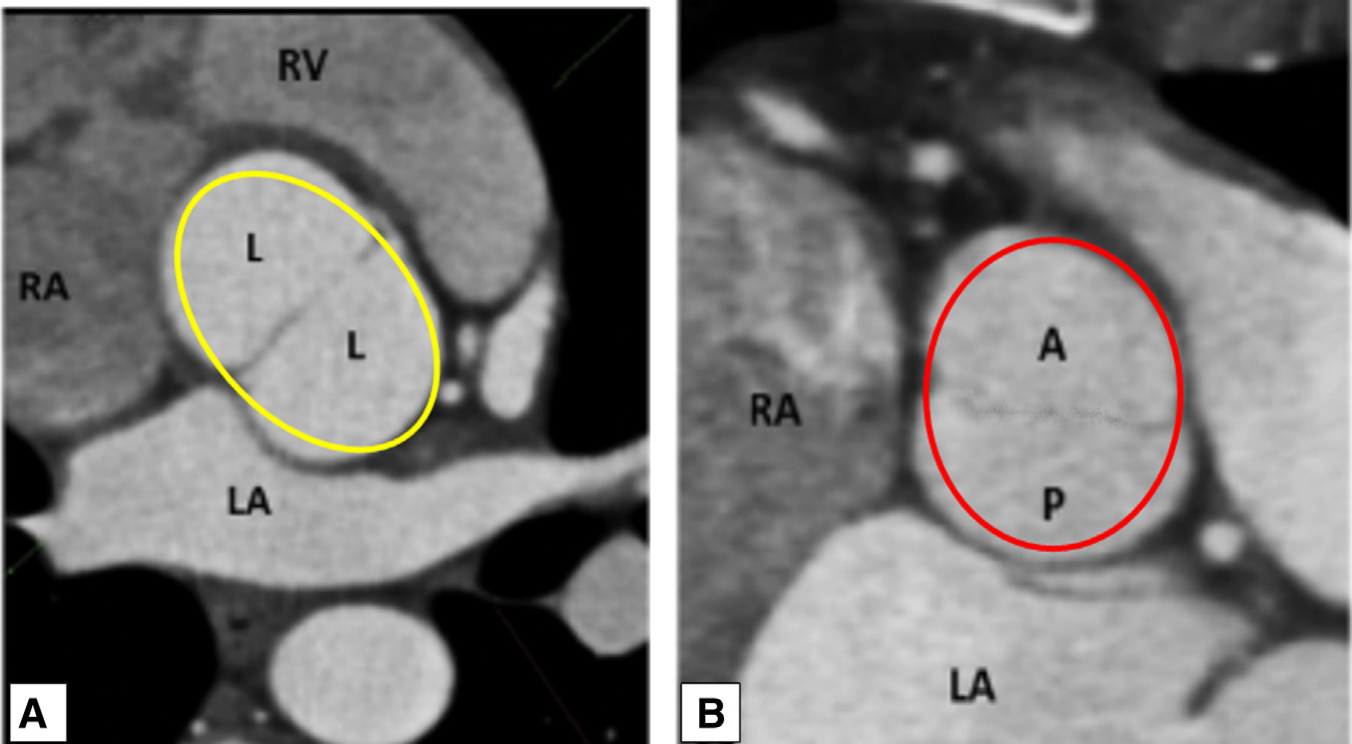
Electrocardiogram-gated cardiac computed tomography (CT) showing 2-sinus BAVs in short axis, in closing position; in the latero-lateral variant (A) the aortic shape is more elliptical (*yellow*) compared with anteroposterior variant (B, *red*). Adapted with permission from Michelena and colleagues.^3^*AP*, Anteroposterior; *LL*, latero-lateral; *RA*, right atrium; *RV*, right ventricle.

Instead, if the valve CO is along the same axis as the aortic dilatation, the ellipsoid will be less pronounced (Figure 1, *B*) and the dilatation will progressively pull on the 2 commissures, stretching on the cusps and their free margins, likely resulting in leaflet free margin elongation. Often, the valve will remain competent or present with trivial regurgitation. When the STJ is replaced with a circular Dacron graft, the distance between the 2 commissures is reduced. The more pronounced the reduction, the more the valve will prolapse, with the consequent risk of valve regurgitation. Plication of both leaflets is then required to restore the correct effective height and valve competence.^2^

The best anatomic condition in which this anatomo-pathological condition can be verified is the 2-sinus BAV,^3^ where we usually have 2 symmetrical leaflets without raphe (type 0 Sievers classification), which can have 2 different CO: latero-lateral and anteroposterior. Progressive dilation of the ascending aorta involving the STJ usually occurs along the anteroposterior axis (toward the great curvature/noncoronary sinus) resulting in an ellipsoidal configuration of the dilated aorta. Therefore, the effect of replacing the ascending aorta with a circular (by definition) Dacron graft will have different effects if the valve has a latero-lateral or an anteroposterior orientation.^4^^,^^5^ In the first case, the new circularity of the STJ will not alter the integrity and function of the valve; on the other hand, in the anteroposterior orientation the 2 commissural posts will be brought closer together and the 2 cusps will inevitably prolapse toward the left ventricle outflow tract, reducing their coaptation. The result is valvular insufficiency. This concept applies when only supracoronary aortic aneurysm is corrected and is even more troublesome because it can cause regurgitation in a previously competent BAV. Fortunately, most BAVs have their orifice oriented along the short axis, because the most common phenotype of 3-sinus BAV is a left-right fusion (a spatial correspondence to the latero-lateral in the 2-sinus BAV). More rarely, we are faced with an anteroposterior configuration (corresponding to the also rare right-non and left-non of the 3-sinus BAV) (Figures 2 and 3).

**Figure 2:**
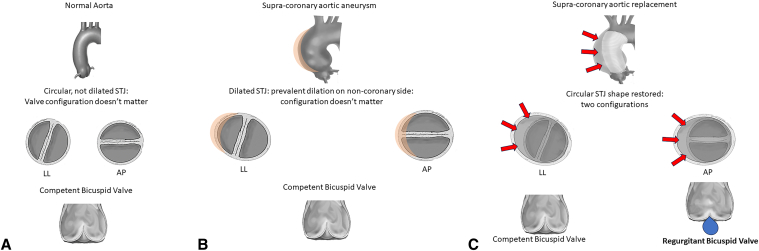
Diagram showing the different orientations of BAV orifice with respect to STJ aspect: Normal (A), dilated/aneurysmatic (B), and after supracoronary aorta replacement (C). *AP*, Anteroposterior; *LL*, latero-lateral; *STJ*, sinotubular junction.

**Figure 3:**
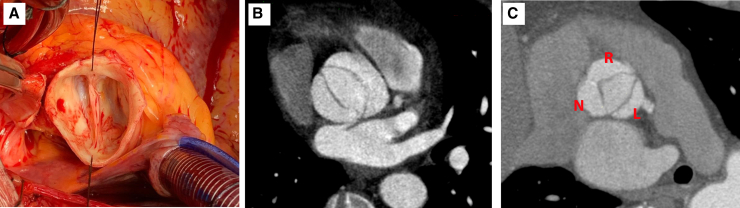
A, Intraoperative view of a 2-sinus BAV with latero-lateral configuration. B, Computed tomography scan view of a 2-sinus BAV, latero-lateral configuration. C, Computed tomography scan view of a raphed BAV with right-non fusion.

Understandably, the size of the aneurysm and the ratio of the aneurysm diameter to the implanted graft may exacerbate this effect: The larger the STJ/graft ratio, the greater the risk of inducing cusp prolapse.

Experienced groups who have published on BAV repair have not analyzed this as a predictor factor of recurrent aortic regurgitation (AR), but given the low incidence of this specific condition and the low incidence of recurrent AR in those studies, it is difficult to prove the concept through normal clinical experience,^6^ and it was not possible to produce a single-center case series. In addition, we need to consider that experienced surgeons will usually correct cusp prolapse promptly.^7^

When addressing a supracoronary aorta replacement, in the presence of these rare valve configurations, surgeons should be aware that some kind of gesture on the cusp will probably be required to correct the induced cusp prolapse and prevent AR. This is particularly important in those patients with nonsignificant preexisting AR. Regardless, CO, although influencing postoperative valve behavior in specific configurations, should not be considered a stand-alone indication for surgical intervention. Established criteria based on aortic dimensions and valve function remain the primary basis for decision-making.

The present article is founded on a mechanistic, anatomic hypothesis, yet it lacks a clinical dataset. It is challenging to prove the concept through normal clinical experience, and it was not possible to produce a single-center case series.

## Conflict of Interest Statement

The authors reported no conflicts of interest.

The *Journal* policy requires editors and reviewers to disclose conflicts of interest and to decline handling or reviewing manuscripts for which they may have a conflict of interest. The editors and reviewers of this article have no conflicts of interest.
